# Design and synthesis of HLA-A*02-restricted Hantaan virus multiple-antigenic peptide for CD8^+^ T cells

**DOI:** 10.1186/s12985-020-1290-x

**Published:** 2020-01-31

**Authors:** Yan Ma, Kang Tang, Yusi Zhang, Chunmei Zhang, Yun Zhang, Boquan Jin, Ying Ma

**Affiliations:** 1 The Fourth Team, Academy of Basic Medicine, the Fourth Military Medical University, Xi’an, 710032 China; 2 Department of Immunology, The Fourth Military Medical University, 169 Changle West Road, Xi’an, 710032 China

**Keywords:** Hantaan virus, Multiple-antigenic peptide, Hemorrhagic fever with renal syndrome, HLA-A*02, Vaccine

## Abstract

**Background:**

Hantaan virus (HTNV) can cause hemorrhagic fever with renal syndrome (HFRS) in humans with severe morbidity and high mortality. Although inactivated HFRS vaccines are given annually for prevention in populations, China still has the highest number of HFRS cases and deaths worldwide. Consequently, vaccination for HFRS requires the development of novel, more effective vaccines. Epitope peptide vaccines have been developed rapidly in recent years and are considered a novel approach for the prevention of infection. Specifically, the multiple antigenic peptide (MAP) design with preferable immunogenicity can arouse a satisfactory immune response for vaccination. However, there are few reports on the design and evaluation of MAP for HTNV.

**Methods:**

Three HLA-A*02-restricted 9-mer cytotoxic T lymphocyte (CTL) epitopes on HTNV glycoprotein and one HLA-A*02-restricted 9-mer CTL epitope on the HTNV nucleocapsid, which have been proven to be immunoprotective in our previous study, were selected for the design of HTNV MAP. A four-branched HTNV MAP was evaluated by the IFN-γ-secreting enzyme-linked immunospot assay and proliferation induction capacity of CD8^+^ T cells and compared with the single HTNV CTL epitope in 17 HLA-A*02^+^ patients with HFRS. The Mann–Whitney *U* test was used for comparison of parameters between different subject groups.

**Results:**

The macromolecular HTNV MAP was designed with a polylysine core and four radially branched single CTL epitope chains. Importantly, HTNV MAP could stimulate CD8^+^ T cell secretion of IFN-γ in HLA-A*02^+^ patients with HFRS. The frequency of IFN-γ-secreting CD8^+^ T cells in the MAP stimulation group was significantly higher than that in the single HTNV CTL epitope stimulation groups (*P* < 0.005). Meanwhile, the activity of IFN-γ-secreting CD8^+^ T cells in the HTNV MAP group was also higher than that of the single CTL epitope groups (*P* < 0.05). Moreover, there was a much stronger ability of HTNV MAP to stimulate CD8^+^ T cell proliferation compared with that of a single HTNV CTL epitope.

**Conclusions:**

The designed HTNV MAP could induce CTL responses ex vivo and may be considered a candidate for the design and development of novel HTNV peptide vaccines.

## Background

Hantavirus (HTV) infection could cause hemorrhagic fever with renal syndrome (HFRS) or hantavirus cardiopulmonary syndrome (HCPS) in humans, which are characterized by an increase in capillary permeability and thrombocytopenia [1]. By the end of 2017, 41 hantavirus species were officially identified by the International Committee on Taxonomy of Viruses (ICTV). HTV can be transmitted via the inhalation of aerosols from rodents such as *Apodemus agrarius* and *Rattus norvegicus* [2]. Recent studies have shown that HTV could also be transmitted through gastrointestinal infections [3]. In Europe and Asia, Hantaan virus (HTNV), Seoul virus (SEOV), Dobrava virus (DOBV), Puumala virus (PUUV) and Saaremaa virus (SAAV) are the major causative agents of HFRS. In Africa, HFRS is mainly caused by Sangassou virus (SANGV). In South America and North America, Andes virus (ANDV), Sin Nombre virus (SNV) and New York virus (NYV) are the most prominent hantaviruses causing HCPS. Approximately 60,000–100,000 cases of HFRS are reported worldwide each year, which affects more than 70 countries, especially the Eurasian developing countries. HTNV is the prototype of HTV and causes severe epidemic HFRS in China. According to the Chinese Center for Disease Control and Prevention (CDC), the incidence of HFRS was 8853 in 2016 in China. With factors such as climate and individual immunity, the fatality rate of HFRS can be up to 15% [4, 5].

The genome of HTNV can be divided into three segments by molecular weight, including the S segment encoding nucleocapsid protein (NP), the M segment encoding Gn and Gc glycoprotein (GP), and the L segment encoding RNA-dependent RNA polymerase (RdRp) [6]. HTNV-NP contains 429–433 amino acids (aa), which have a highly conserved region for the B cell epitope [7]. Moreover, there were also reports on the identification of T cell epitopes from HTNV-NP, which was one of the focuses of our study [7–9]. HTNV-GP contains 1132–1184 aa, which could mature into HTNV-Gn and HTNV-Gc through cleavage [10]. Many published data suggest that HTNV-NP could induce both cellular and humoral immunity in the body, while HTNV-GP is mainly considered a humoral immunogen that induces neutralizing antibodies [5]. However, Terajima et al. identified an HLA-A*24-restricted 9-mer epitope on HTNV-Gc protein and established the epitope-specific cytotoxic T lymphocyte (CTL) cell line [11]. Ma et al. identified one HTNV-GP-derived H2-K^b^-restricted CTL epitope in C57BL/6 mice [12]. Importantly, in our previous studies, we identified seven HTNV-GP-derived HLA-A*02-restricted CTL epitopes that could induce specific protective CTL immune responses in patients with HFRS [13]. These results suggested that HTNV-GP could also induce a specific T cell response in HTNV infection.

Currently, symptomatic and supportive therapies are mainly used for the treatment of HFRS after HTNV infection in the clinic when the disease progresses rapidly [14]. Vaccination is an effective and economic way to prevent viral infection in the population. Currently, vaccine developments for hantavirus diseases include inactivated vaccines, virus-like particle vaccines, recombinant proteins, virus-vectored recombinant vaccines and nucleic acid-based molecular vaccines [15–17]. In China, the inactivated HFRS vaccine has been widely used for inoculation of the population for many years. Although the HFRS inactivated vaccines could reduce the incidence rate of HFRS, the immunogenicity of such vaccine is limited, with poor induction of protective antibodies and cellular immunity [18]. Recently, Jung J et al. found that the inactivated HFRS vaccine had only moderate effectiveness in high-risk populations residing in endemic areas [19]. Therefore, the demand for novel and more effective HFRS vaccines has become urgent. The polypeptide vaccine has the advantages of high specificity, safety and easy preservation, which could enhance immune response though linear concatenation of multi-antigen epitopes or multiple-antigenic peptide (MAP) dimensional structure [20]. A recent study has shown that the MAP vaccine for *toxoplasma gondii* could protect mice against acute and chronic toxoplasmosis [21]. In dengue virus infection, MAP was expected to be used for the diagnosis of dengue infection by detecting anti-MAP immunoglobulin M (IgM) and IgG in the serum of patients [22]. Moreover, a thymus-dependent antigen designed as the MAP structure could effectively induce T cell responses, thereby protecting mice from *Staphylococcus aureus (S. aureus)* infection [23]. Based on these studies, MAP design with preferable immunogenicity can arouse a strong immune response against infection. However, there is scarce research on the design and evaluation of the effects of HTNV polypeptide vaccines, especially MAP for HTNV.

Notably, epitopes inducing cellular immune responses in vivo are restricted by various HLA alleles in humans. In the Chinese Han population, HLA-A*02 has the widest distribution and the highest frequency allele among HLA class I alleles [24]. Therefore, the identification of the protective HTNV CTL epitopes restricted by HLA-A*02 has significant value not only for the exploration of HTNV-specific CTL immune responses but also for the design of HTNV polypeptide vaccines. Our previous studies identified seven HLA-A*02-restricted HTNV-Gn/Gc CTL epitopes and one HLA-A*02-restricted HTNV-NP CTL epitope [13, 25]. We also confirmed that HLA-A*02-restricted HTNV CTL epitopes could induce protective IFN-γ secretion in patients with HFRS and inhibit HTNV replication in HLA-A2.1/K^b^ transgenic mice [13, 26]. Therefore, these HLA-A*02-restricted protective CTL epitopes could be considered candidates for the design of novel HTNV polypeptide vaccines.

In this study, we selected four HLA-A*02-restricted HTNV 9-mer CTL epitopes, which have been proven to have immunoprotective effects against HTNV infection, and designed a MAP structure polypeptide of HTNV. We further compared the immune effects of the designed HTNV MAP with a single HTNV CTL peptide and verified the ability of HTNV MAP to induce effective CD8^+^ T cell responses ex vivo. This study may provide an important basis for the development of novel effective peptide vaccines for HFRS.

## Materials and methods

### Patients

Forty-five patients with HFRS infected with HTNV were enrolled in the study at the Department of Infectious Diseases at Tangdu Hospital of the Fourth Military Medical University (Xi’an, China). HTNV infection was confirmed by the detection of HTNV-specific IgM or IgG antibodies in serum specimens. According to clinical observation, the course of HFRS disease can be divided into five stages: fever, hypotension, oliguric, diuretic and convalescence stages [13, 26]. In order to facilitate experimental research, the disease stages were generally divided into the acute phase (including fever, hypotension and oliguric stages) and convalescence phase (including diuretic and convalescence stages). According to the Ministry of Health of the People’s Republic of China “HFRS Prevention Strategy”, HFRS disease severity could be classified into four clinical types: (1) mild: mild renal failure without an obvious oliguric stage; (2) moderate: obvious symptoms of uremia, effusion (bulbar conjunctiva), hemorrhage (skin and mucous membrane), and renal failure with a typical oliguric stage; (3) severe: severe uremia, effusion (bulbar conjunctiva and either peritoneum or pleura), hemorrhage (skin and mucous membrane), renal failure with oliguria (urine output, 50–500 mL/day) for 5 days or anuria (urine output < 50 mL/day) for 2 days; and (4) critical: for those with more than one of the following symptoms during severe disease, such as refractory shock, visceral hemorrhage, heart failure, pulmonary edema, brain edema, severe secondary infection, and severe renal failure with either oliguria (urine output 50–500 mL/day) for > 5 days or anuria (urine output < 50 mL/day) [13, 26]. We have excluded patients with other kidney diseases, diabetes, cardiovascular diseases, autoimmune diseases, hematological diseases, viral hepatitis and other liver diseases.

### Sample collection

Peripheral blood samples of patients with HFRS were collected in the acute phase and convalescence phase, respectively. Ten milliliters of peripheral blood from each patient with HFRS was sterile extracted with ethylene diamine tetraacetic acid (EDTA) anticoagulant. After centrifuging at 3000 RPM for 10 min, the upper plasma was packaged and preserved at − 80 °C. Peripheral blood mononuclear cells (PBMCs) were isolated by standard Ficoll-Hypaque density gradient centrifugation from anticoagulant peripheral blood and were resuspended with 10% fetal calf serum (FCS) for later experiments or frozen in liquid nitrogen (90% FCS, 10% dimethyl sulfoxide) for reserve. Meanwhile, the medical record number, general clinical information, clinical symptoms, biochemical examination results and treatment plan of each patient were recorded in detail.

### Identification of HLA-A*02^+^ individuals

HLA-A*02^+^ patients with HFRS were screened by flow cytometry (FCM). Briefly, 5 × 10^5^ PBMCs of each patient with HFRS were washed and combined with mouse anti-human HLA-A*02-PE monoclonal antibody (mAb) (5 μL/test) (Biolegend, San Diego, CA, USA, clone: BB7.2). Meanwhile, the isotype control tube contained mouse IgG1-PE (5 μL/test) (Biolegend, San Diego, CA, USA). After incubation at 4 °C for 30 min, the PBMCs were washed and mixed gently. To each tube was added 300 μL of FCM fix solution, and samples were detected by flow cytometry (FACScan; BD Biosciences, San Jose, CA, USA). HLA-A*02^+^ PBMCs were selected and applied in the following assays.

### Design, synthesis and purification of HTNV MAP

Based on the ability to induce HTNV epitope-specific protective immune responses, four HLA-A*02-restricted HTNV-NP and Gn/Gc CTL epitopes identified in our previous studies were selected for the design of HTNV MAP. The amino acid sequences of each epitope are shown in Table 1 [13, 25, 26]. The MAP molecule always consists of a polylysine (Lys) core with symmetrically branched oligopeptide chains. Each oligopeptide is typically 10–20 amino acids in length and is attached radially to the polylysine core. In our study, the four selected HLA-A*02-restricted HTNV-NP and Gn/Gc CTL epitopes constitute the oligopeptide chains of MAP. Since MAP usually has a large molecular weight, the quality control measure of high-performance liquid chromatography (HPLC) is difficult to implement. Therefore, we used indirect methods to synthesize MAP. Each 9-mer CTL epitope peptide was first synthesized, purified and analyzed by mass spectrometry or HPLC. Then, the synthesized 9-mer peptides were linked to the polylysine core by cysteine (Cys). We commissioned Synpeptide Co. Ltd. (Nanjing, China) to synthesize, purify and identify the HTNV MAP macromolecule.

**Table 1:** Detailed information of the selected HLA-A*02-restricted HTNV CTL epitopes

HTNV structure protein	Sequence	Start aa	End aa	Abbreviation
GP	VMASLVWPV	8	16	VV9
GP	SLTECPTFL	996	1004	SL9
GP	LIWTGMIDL	358	366	LL9
NP	FVVPILLKA	129	137	FA9

*GP* glycoprotein, *NP* nucleocapsid protein

### Immunomagnetic bead removal of CD4^+^ T cells from PBMCs

The Dynabeads® human CD4^+^ T cells positive isolation kit (Thermo Fisher Scientific, Waltham, MA, USA) was used to remove CD4^+^ T cells from PBMCs. In brief, after washing the dynabeads, the beads were transferred to a tube with the PBMCs resuspended in buffer 1 (25 μL dynabeads/1 × 10^7^ PBMCs/1 mL buffer 1). Then, the samples were incubated for 20 min at 4 °C with gentle tilting and rotation. Next, the tube was placed in a magnet for 2 min. While the tube was still in the magnet, the supernatant was carefully collected. CD4^+^ T cells were left in the tube, and the supernatant mainly contained the CD4^+^ T cell-deleted PBMCs, including CD8^+^ T cells, antigen-presenting cells and other cells.

### Ex vivo IFN-γ enzyme-linked immunospot (ELISPOT) assay

CD4^+^ T cell-deleted PBMCs of HLA-A*02^+^ patients with HFRS were used for IFN-γ ELISPOT detection. The human IFN-γ ELISPOT kit (DAKEWE Biotech Company, China) was used to detect the capacity of IFN-γ production by CD8^+^ T cells stimulated with a single HTNV CTL epitope or HTNV MAP. Briefly, 5 × 10^5^ CD4^+^ T cell-depleted PBMCs in RPMI 1640 containing 10% FCS were seeded in each well of 96-well plates precoated with anti-human IFN-γ mAb (1-D1K). Meanwhile, the cells were stimulated with single 9-mer peptide (80 μmol/L), peptide mixture (each single peptide concentration is 80 μmol/L) or MAP (80 μmol/L). After 42 h of incubation, the cells were removed by emptying the plate and washed with phosphate buffer solution (PBS). Then, 100 μL of biotinylated secondary anti-human IFN-γ antibody (7-B1–6-biotin) was added to each well for a 1-h incubation at 37 °C. After washing, 100 μL of streptavidin-horseradish peroxidase (HRP) was added to each well for a 1-h incubation at 37 °C. Then, 100 μL of AEC solution was added to each well after washing. The color development was stopped by washing extensively. Cells with 10 μg/mL phytohemagglutinin (PHA) (DAKEWE Biotech Company, China) or with no peptide stimulation were used as positive or negative controls, respectively. The spots representing peptide-specific IFN-γ-producing CTLs were counted using an automatic ELISPOT reader (Cellular Technology Limited, USA). Adjusted spot-forming cells (SFCs) after subtracting negative values are expressed as SFCs/10^6^ cells.

### CFSE staining for proliferation assay

To evaluate the capacity of specific CTL proliferation, 2 × 10^7^/mL PBMCs from HLA-A*02^+^ patients with HFRS were incubated with 10 μmol/L 6-carboxyfluorescein succinimidyl ester (CFSE, Molecular Probes, OR) for 15 min at 37 °C and kept away from light. The staining was terminated upon the addition of an equal volume of fetal bovine serum (FBS) for 5 min at room temperature. Then, the cells were washed twice with PBS and stimulated with HTNV single 9-mer epitope peptide or HTNV MAP (20 μmol/L). Staphylococcal enterotoxin B (SEB, 200 ng/mL, Sigma-Aldrich, MO, USA) stimulation of PBMCs served as positive controls. After 2 days of culture, 10% exogenous IL-2 was added. After 5 days, cells were harvested and stained with anti-CD3-PerCP-Cy5.5 mAb (Biolegend, San Diego, CA, USA) and anti-CD8-PE mAb (Biolegend, San Diego, CA, USA). Flow cytometry analysis for CFSE positive peak position and peak shape was then performed.

### Statistical analysis

The data were statistically analyzed by SPSS 16.0 (SPSS Inc.; Chicago, IL, USA) and GraphPad Prism software, version 6 (GraphPad; La Jolla, CA, USA). The Mann–Whitney *U* test was used for comparison of parameters between different subject groups. The parameter data are expressed as medians with corresponding interquartile ranges (IQRs). *P* < 0.05 was considered statistically significant.

## Results

### HTNV MAP structure design

**Fig. 1:**
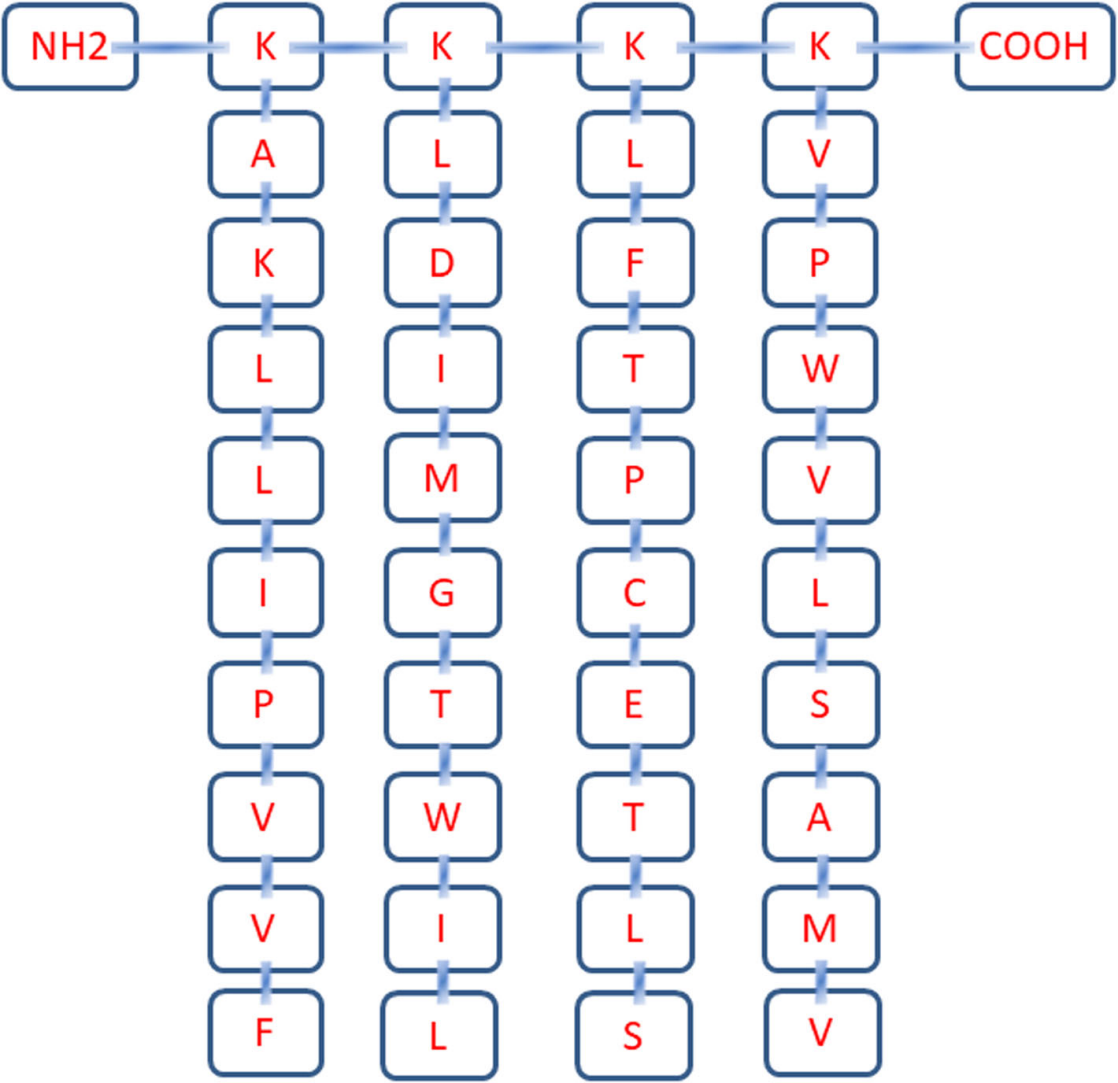
The design and synthesis of the HTNV MAP. Four-branched HTNV MAP consists of a polylysine core and attached four HLA-A*02 restricted HTNV 9-mer CTL epitopes. The structure from the amino terminal to the carboxyl terminal is shown. K, lysine; A, alanine; L, leucine; I, isoleucine; P, proline; V, valine; F, phenylalanine; D, aspartic acid; M, methionine; G, glycine; T, tryptophan; C, cysteine; E, glutamic acid; S, serine

Based on our previous results, three immunoprotective HLA-A*02-restricted HTNV-Gn/Gc CTL epitopes, namely, VV9, SL9 and LL9, and one immunoprotective HLA-A*02-restricted HTNV-NP CTL epitope, FA9, were selected for use as branched oligopeptide chains in the design of HTNV MAP. Detailed information on the HTNV CTL epitopes is shown in Table 1. Specifically, the MAP was designed with a main chain of polylysine as a core and four HTNV 9-mer linear CTL epitopes as branched peptides. Therefore, the MAP showed an outer layer consisting of branched peptides ligated to the polylysine core at the C-terminus. The polylysine core portion functions to link the branched peptides to form the MAP structure macromolecule. The molecular weight of the synthesized HTNV MAP is 4530.7 kDa with 40 amino acids and greater than 90% purity. The details of the structure of the designed HTNV MAP are shown in Fig. 1. The four-branched HTNV MAP (MAP4) with a unique spatial conformation was then investigated for its ability to induce specific CTL responses.

### HTNV MAP could stimulate a higher frequency of CD8^+^ T cell responses compared with a single CTL epitope in HLA-A*02^+^ patients with HFRS

**Fig. 2:**
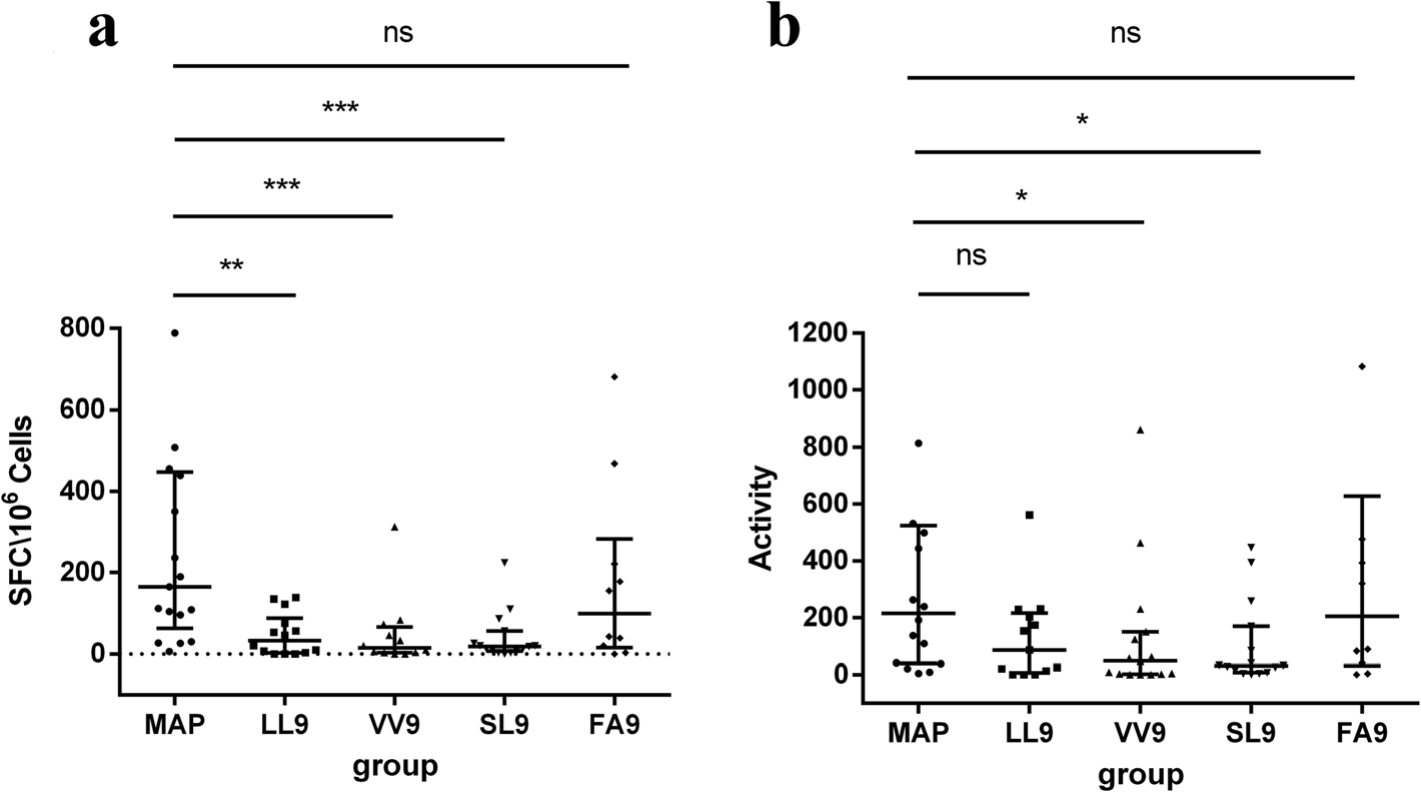
Comparison of IFN-γ-secreting CD8^+^ T cell frequency and activity after HTNV MAP and single-peptide stimulation. IFN-γ-secreting cell frequency was detected by enzyme-linked immunospot (ELISPOT) assay. CD4^+^ T cell-depleted PBMCs from HLA-A*02^+^ patients with HFRS were stimulated with single peptide (80 μmol/L), MAP (80 μmol/L) or phytohemagglutinin (10 μg/mL). HTNV single CTL epitope LIWTGMIDL, VMASLVWPV, SLTECPTFL, and FVVPILLKA are represented by LL9, VV9, SL9, and FA9, respectively. **a** The frequency of IFN-γ-secreting CD8^+^ T cells was detected. All cell frequencies were converted to the number of spot-forming cells per 1 × 10^6^ CD4^+^ T cell-depleted PBMCs (SFCs/10^6^ cells). **b** The activity of IFN-γ-secreting CD8^+^ T cells was measured. The activity was evaluated as the average of the spot size and intensity for each well. Each black dot represents one patient sample. The Mann-Whitney *U* test was used to determine the difference between two groups. The black line indicates the median and corresponding interquartile range (IQR). *P < 0.05, **P < 0.01, ***P < 0.001, ns indicates no significant difference

To evaluate the ability of the designed HTNV MAP to stimulate CTL responses ex vivo, we first stimulated the CD4^+^ T cell-depleted PBMCs of HLA-A*02^+^ patients with HFRS with HTNV MAP and four other single HTNV CTL epitopes (SL9, VV9, LL9 and FA9). The frequency of cells secreting IFN-γ was detected by ELISPOT. The results showed that both HTNV MAP and each HTNV CTL epitope could stimulate the CD8^**+**^ T cells of HLA-A*02^+^ patients with HFRS to produce IFN-γ ex vivo. Then, we compared the frequencies of IFN-γ-secreting cells between MAP stimulation and other single CTL epitope stimulation groups. Notably, there were 165 (43–447) SFCs/10^6^ cells for HTNV MAP, 33 (2–88) SFCs/10^6^ cells for epitope LL9, 15 (3–66) SFCs/10^6^ cells for epitope VV9, 18 (7–56) SFCs/10^6^ cells for epitope SL9, and 99 (15–282) SFCs/10^6^ cells for epitope FA9. The frequency of IFN-γ-secreting CD8^+^ T cells in the HTNV MAP stimulation group was significantly higher than that in the LL9, VV9 and SL9 peptide stimulation groups (*P* < 0.005) (Fig. 2a). Although there was no statistically significant difference in the frequency of IFN-γ-secreting CD8^+^ T cells between the HTNV MAP group and the single epitope FA9 stimulation group (*P* > 0.05), the median frequency of CD8^+^ T cells stimulated by HTNV MAP was still higher than that of epitope FA9 (Fig. 2a). The results suggested that HTNV MAP could induce stronger CD8^+^ T cell responses than the single HTNV CTL LL9, VV9 and SL9 epitopes. Meanwhile, the activity of CD8^+^ T cells secreting IFN-γ was analyzed, which was characterized as the average spot size and intensity for each well. Importantly, the CD8^+^ T cell activity was 214.50 (38.75–523.00) in the HTNV MAP stimulation group, 86.00 (6.50–215.50) in the epitope LL9 group, 48.00 (2.00–150.00) in the epitope VV9 group, 32.00 (8.00–169.00) in the epitope SL9 group, and 204.50 (31.50–627.50) in the epitope FA9 group. The activity of IFN-γ-secreting CD8^+^ T cells in the HTNV MAP group was significantly higher than that in the single epitope VV9 and SL9 groups (*P* < 0.05) (Fig. 2b).

### HTNV MAP could induce stronger CD8^+^ T cell proliferation than a single HTNV CTL epitope in HLA-A*02^+^ patients with HFRS

**Fig. 3:**
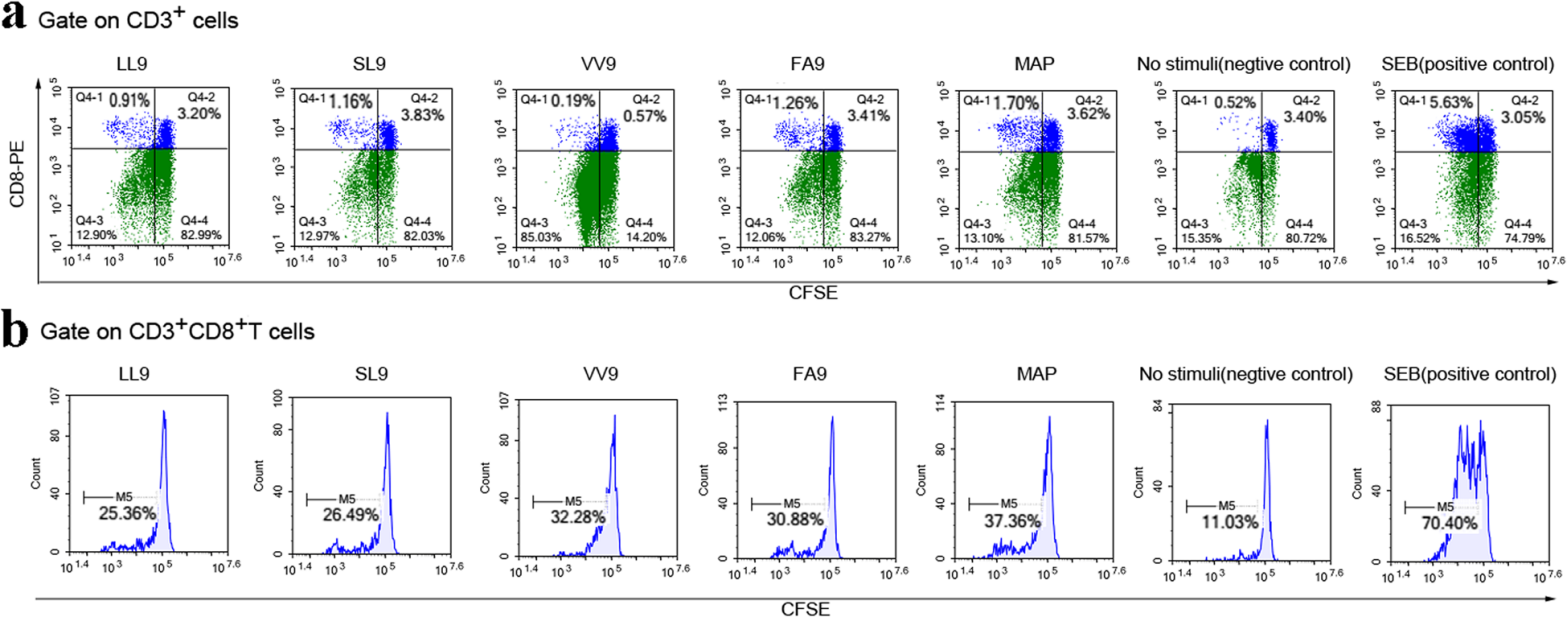
Representative CFSE staining of CD8^+^ T cell proliferation stimulated with HTNV MAP and single peptide. Isolated PBMCs from HLA-A*02^+^ patients with HFRS were labeled with CFSE (5 μM) and stimulated with HTNV MAP or single epitope LL9, VV9, SL9 and FA9. The cells were stained with anti-CD3 PerCP-Cy5.5 and anti-CD8 PE mAb after 5 days of culture. **a** The scatter plot for the flow cytometry analysis of cell proliferation from one representative HLA-A*02^+^ HFRS patient after stimulation with different groups of stimuli when gated on CD3^+^ cells. The upper left quadrant shows that the CFSE fluorescence intensity was reduced in cells representing the proliferation percentages. The number indicates the percentage of cells in the boxed area. **b** The histogram shows the peak shift of the CFSE^dim^CD3^+^CD8^+^T cells in different stimulation groups. The degree of shift to the left of the curve reflects the proliferation percentage of CD8^+^ T cells. The SEB stimulation group was used as a positive control, and the no stimulation group was used as a negative control

The PBMCs isolated from HLA-A*02^+^ patients with HFRS were stained with CFSE to perform a proliferation assay. The designed HTNV MAP and four other single HTNV CTL epitopes (SL9, VV9, LL9 and FA9) were used as stimuli to induce the expansion of CD8^+^ T cells. The SEB stimulation group was used as a positive control. Cell proliferation was calculated as a percentage of CFSE fluorescence reduction. After 5 days of stimulation, the CD8^+^ T cells in the HTNV MAP group and each single CTL epitope group showed different levels of proliferation. As shown in a representative patient (Fig. 3a), the percentage of CFSE^dim^ CD8^+^ T cells in SEB stimulation was higher than that in HTNV MAP and other single CTL epitope stimulation groups, and the percentage of CFSE^dim^ CD8^+^ T cells in the HTNV MAP stimulation group was higher than that in the other four single CTL epitope stimulation groups (Fig. 3b). Thus, the designed HTNV MAP may have a stronger capacity to induce the proliferation of CD8^+^ T cells than the single HTNV CTL epitope in patients with HLA-A*02^+^ HFRS.

## Discussion

Inactivated HFRS vaccines have been widely used in Asia for population vaccination. However, it still could not completely prevent HTNV infection in humans. Based on our previously identified HTNV structure protein CTL epitopes restricted by HLA-A*02 with proven protective effects, we designed HTNV MAP with a polylysine core and four radially attached HTNV 9-mer CTL epitope oligopeptide chains. Here, we demonstrated that the designed HTNV MAP could stimulate IFN-γ secretion from CD8^+^ T cells and induce strong proliferation of CD8^+^ T cells derived from HLA-A*02^+^ patients with HFRS ex vivo, suggesting HTNV MAP could effectively stimulate HTNV-specific CD8^+^ T cell of patients in vitro. The results may lay an important foundation for the development of the novel HTNV peptide vaccine.

HTNV-NP, Gn and Gc have been shown to be strongly immunogenic, but the specific immune responses to HTNV-NP and GP are not completely consistent among different individuals. We designed the MAP macromolecule of HTNV to stably combine the four single CTL epitopes, which may enhance immunogenicity by increasing the molecular weight of the antigen. Previous studies have shown that MAP could retain all of the immunological properties as an alternative antigen, which could induce a strong immune response against viral infection [27, 28]. In fact, MAP has been widely used for vaccine design in a variety of infectious diseases. Most studies applied a tetramer or octamer structure for MAP design [21, 27, 28]. In our previous study, we identified seven HLA-A*02-restricted HTNV-GP CTL epitopes and found that higher frequencies of epitope-specific CTLs were associated with milder disease severity of HFRS. Among the seven epitopes, three epitopes, namely, VV9, SL9, and LL9, showed higher HLA-A*02-binding affinity and could induce higher frequencies of epitope-specific CTLs in patients with HFRS compared with other CTL epitopes. Therefore, we selected the three epitopes VV9, SL9, and LL9 for immunization of the HLA-A2.1/K^b^ transgenic mice. Epitope VV9, SL9, and LL9 vaccinations all exhibited significant inhibition of HTNV replication in HLA-A2.1/K^b^ transgenic mice [13]. Moreover, we have also demonstrated that HTNV-NP-derived CTL epitope FA9 could mediate effective protective responses to inhibit HTNV replication in HLA-A2.1/K^b^ transgenic mice [26]. Hence, we chose the four HTNV CTL epitopes VV9, SL9, LL9, and FA9, which could induce specific CTL responses to inhibit HTNV replication in HLA-A2.1/K^b^ transgenic mice, to synthesize HTNV MAP.

We employed a tetramer to design the 4-branched HTNV MAP, which may have an appropriate spatial position of the four different HTNV 9-mer CTL epitopes from each other. Moreover, the 4-branched CTL epitopes attached to a polylysine core could form high stability in the body, which is not easily degraded. Elucidation of the specific response characteristics induced by MAP may provide an important experimental basis for the effectiveness evaluation of the MAP design. In our study, the constructed 4-branched HTNV MAP could stimulate high-level IFN-γ secretion of CTL cells derived from HLA-A*02^+^ patients with HFRS ex vivo. Notably, the frequency of MAP-stimulated IFN-γ-secreting CD8^+^ T cells was significantly higher than that of LL9, VV9 and SL9 single epitope stimulation. For the epitope FA9, enlarging the sample size may increase the differences between HTNV MAP and FA9. These results indicated that the designed HTNV MAP had a better ability than the LL9, VV9 and SL9 single epitopes to stimulate CD8^+^ T cell responses ex vivo. In fact, IFN-γ is one of the key cytokines for anti-HTNV infection responses, which could promote the processing and presentation of the MHC-peptide complex and upregulate the expression of MHC class I molecules on the cell surface [29]. Therefore, we speculated that the designed HTNV MAP could induce a stronger immune response against HTNV infection than other single peptides in HLA-A*02^+^ patients with HFRS.

Moreover, HTNV MAP is likely more potent than other single peptides in stimulating proliferation of CD8^+^ T cell derived from HLA-A*02^+^ patients with HFRS. It is generally believed that the T cell immune responses play an important anti-virus effect after HTNV infection. In patients in the early stage of severe or critical HFRS, HTNV-specific CTL responses were impaired, suggesting that early CTL response disability may be the main cause of exacerbation of HFRS [30]. In the mouse model of HTNV persistent infection, few effector CTLs were detected, but a large number of effector CTLs were present in a mouse model of transient HTNV infection, indicating that effector CTLs are important for clearance of HTNV [31, 32]. Additionally, neutralizing antibodies could bind to HTNV and block HTNV entry into cells. However, neutralizing antibodies could not clear intracellular HTNV infection. HTNV infection was maintained in the presence of neutralizing antibodies, indicating that neutralizing antibodies alone cannot eliminate HTNV. Notably, HTNV NP was undetectable after adoptive transfer of HTNV-specific CTLs into HTNV-infected severe combined immunodeficient mice, suggesting that HTNV-specific CTLs are the major effector cells against intracellular HTNV infection and contribute to the clearance of HTNV [31, 33]. Less or functionally defective early stage HTNV-specific CTLs will lead to viral replication and spread, aggravating the disease and even causing it to become life-threatening. The HTNV MAP demonstrated the capability of IFN-γ production and proliferation of CD8^+^ T cells, which may support the rational design of novel candidate vaccines for HTNV infections.

## Conclusions

In this study, we selected three HLA-A*02-restricted HTNV-GP 9-mer CTL epitopes and one HLA-A*02 restricted HTNV-NP CTL epitope and designed a 4-branched HTNV MAP structure polypeptide. Importantly, we found that the HTNV MAP could stimulate IFN-γ production and proliferation of the specific CD8^+^ T cells of HLA-A*02^+^ patients with HFRS ex vivo, which may provide the basis for the development of a novel HTNV peptide vaccine. Since stimulating PBMCs from convalescent patients does not necessarily correlate to vaccine immunogenicity, our data could not prove that MAP is more immunogenic than the single HTNV CTL peptide. In the future, we will increase the sample size and focus on MAP-induced CTL responses, including the frequency and responses of epitope-specific CTLs in HLA-A2.1/K^b^ transgenic mice after MAP vaccination, and the comparison of the protective efficacy between MAP and single peptide vaccination to verify the immunogenicity and immunoreactivity of HTNV MAP in vivo.

## Data Availability

All data generated or analyzed during this study are included in this published article.

## Abbreviations

ANDV: Andes virus
CFSE: 6-carboxyfluorescein succinimidyl ester
CTL: Cytotoxic T lymphocyte
DOBV: Dobrava virus
ELISPOT: Enzyme-linked immunospot assay
FA9: FVVPILLKA
FCM: Flow cytometry
FCS: Fetal calf serum
GP: Gn and Gc glycoprotein
HCPS: Hantavirus cardiopulmonary syndrome
HTNV: Hantaan virus
HTV: Hantavirus
ICTV: International Committee on Taxonomy of Viruses
Ig: Immunoglobulin
IQR: Interquartile range
LL9: LIWTGMIDL
MAP: Multiple-antigenic peptide
NP: Nucleocapsid protein
NYV: New York virus
PHA: phytohemagglutinin
PUUV: Puumala virus
RdRp: RNA-dependent RNA polymerase
SAAV: Saaremaa virus
SANGV: Sangassou virus
SEOV: Seoul virus
SFC: Spot-forming cell
SL9: SLTECPTFL
SNV: Sin Nombre virus
VV9: VMASLVWPV
